# Autologous keratinocyte suspension in platelet concentrate accelerates and enhances wound healing – a prospective randomized clinical trial on skin graft donor sites: platelet concentrate and keratinocytes on donor sites

**DOI:** 10.1186/1755-1536-6-8

**Published:** 2013-04-09

**Authors:** Samia Guerid, Salim E Darwiche, Mette M Berger, Lee Ann Applegate, Messod Benathan, Wassim Raffoul

**Affiliations:** 1 Plastic and Reconstructive Surgery Service, Centre Hospitalier Universitaire Vaudois, Rue du Bugnon 46, Lausanne 1011, Switzerland; 2 Department of Intensive Care Medicine, Centre Hospitalier Universitaire Vaudois, Rue du Bugnon 46, Lausanne 1011, Switzerland; 3 Unit of Regenerative Therapy, Plastic and Reconstructive Surgery, Department of Musculoskeletal Medicine, Centre Hospitalier Universitaire Vaudois, CHUV/UNIL, EPCR-02, Epalinges 1066, Switzerland

## Abstract

**Background:**

Wound healing involves complex mechanisms, which, if properly chaperoned, can enhance patient recovery. The abilities of platelets and keratinocytes may be harnessed in order to stimulate wound healing through the formation of platelet clots, the release of several growth factors and cytokines, and cell proliferation. The aim of the study was to test whether autologous keratinocyte suspensions in platelet concentrate would improve wound healing. The study was conducted at the Lausanne University Hospital, Switzerland in 45 patients, randomized to three different topical treatment groups: standard treatment serving as control, autologous platelet concentrate (PC) and keratinocytes suspended in autologous platelet concentrate (PC + K). Split thickness skin graft donor sites were chosen on the anterolateral thighs of patients undergoing plastic surgery for a variety of defects. Wound healing was assessed by the duration and quality of the healing process. Pain intensity was evaluated at day five.

**Results:**

Healing time was reduced from 13.9 ± 0.5 days (mean ± SEM) in the control group to 7.2 ± 0.2 days in the PC group (*P* < 0.01). An addition of keratinocytes in suspension further reduced the healing time to 5.7 ± 0.2 days. Pain was reduced in both the PC and PC + K groups. Data showed a statistically detectable advantage of using PC + K over PC alone (*P* < 0.01).

**Conclusion:**

The results demonstrate the positive contribution of autologous platelets combined with keratinocytes in stimulating wound healing and reducing pain. This strikingly simple approach could have a significant impact on patient care, especially critically burned victims for whom time is of the essence.

**Clinical trial registry information:**

Protocol Record Identification Number: 132/03

Registry URL: http://www.clinicaltrials.gov

## Background

Despite the progress achieved in the past few decades, wound healing remains a difficult issue to which modern medicine does not always have an efficient response. The surgical treatment of wounds focuses primarily on accelerating the healing process and overcoming the initial danger and deleterious consequences associated with bleeding and infection. However, under certain circumstances, surgery is not an option. As such, methods like hemotherapy, which consists of applying dressings soaked in blood to the wound itself, have been applied in order to accelerate the healing of certain difficult wounds. Physicians who used this treatment were unaware that they were in fact treating wounds with growth factors [1,2].

In recent years, new treatments focusing on dressing design and composition have been suggested to accelerate wound healing. The ideal dressing should be easy to use, allow the absorption of blood and secretions, reduce local pain and permit a pain-free dressing replacement [3,4]. Affordability is also an important factor. Novel ‘smart’ dressings have indeed been designed to cover graft donor sites and stimulate their healing time. However, none satisfies all the criteria mentioned above, hence the importance of researching other tools. Specific growth factors such as platelet-derived growth factor (PDGF), epidermal growth factor (EGF) and fibroblast growth factor (FGF) have been used in topical treatments [5] but results published thus far are inconclusive, probably because of the complexity of the healing phenomenon which cannot be stimulated by the local application of one factor at any given time, especially since the dosages are often well above physiological levels. Indeed, the orchestrated release of growth factors must be respected in order to best emulate the physiological process [6-8]. Therefore, even with increased precision in surgical technique, post-operative smart dressings, administration of growth factors and regular use of nutritional supplements, reported healing time and risk of complications remained unchanged [9,10], resulting in inconsistent and disappointing outcomes with dissuasive treatment costs.

Platelets play a fundamental role in wound healing, primarily through the formation of platelet clots and the release of many growth factors and cytokines [5]. It has also been shown that epidermal keratinocytes play an essential role in wound healing [11-17]. Keratinocytes, when applied to a wound, should theoretically induce and stimulate healing by releasing growth factors or multiplying *in situ*[18], thereby enhancing the healing effect of blood platelets. Theoretically, it is possible to amplify the effects of the first steps of the wound healing cascade by increasing the local concentration of platelets and keratinocytes which would then induce an increased production of trophic and growth factors [19-23].

A prospective randomized study with three groups of patients was performed in order to test whether a platelet concentrate or keratinocytes suspended in a platelet concentrate could enhance wound healing. One experimental group was treated with an autologous platelet concentrate, another experimental group received a suspension of autologous keratinocytes combined with a platelet concentrate while a standard treatment was administered to patients in the control group.

## Methods

### Study design

The clinical study was conducted as a prospective, randomized controlled, blinded phase I trial (Protocol Record 132/03; http://www.clinicaltrials.gov). After approval by the institutional ethics committee and the Swiss agency for therapeutic products (Swissmedic), 45 patients aged between 18 and 80 years and presenting graft donor sites not exceeding 15% of their total body surface were enrolled in the study between June 2005 and March 2006 at the Lausanne University Hospital in Switzerland. Each collected skin graft was 0.2 mm in thickness, located on the anterolateral region of the thigh and corresponding to a mean area of 180cm^2^ (range 25 to 200 cm^2^). Skin grafts were collected by means of a dermatome (Aesculap AG, Tuttlingen, Germany) with a thickness of 0.2 mm, which is the standard in our department in burn patients in order to achieve healing. In order to create comparable wounds, we used the same protocol in all patients. Exclusion criteria were: treatment with immunosuppressors or corticoids, terminal renal insufficiency and severe peripheral arteriopathy. Patients were randomized to one of three groups of 15 patients: control, platelet concentrate (PC) and platelet concentrate with keratinocytes (PC + K) (Table 1). Block randomization was generated using a computer program (S-Plus 4.0 for Windows, Microsoft Schweiz, Richtistrasse 3, 8304 Wallisellen (Zurich), Switzerland) by an investigator with no clinical involvement in the trial. Allocation was concealed from patients and observers.

**Table 1:** Patient population characteristics

**Group**	**Control**	**PC**	**PC + K**
	**(n = 15)**	**(n = 15)**	**(n = 15)**
Age in years, mean ± SEM (median)	42.5 ± 3.1 (39)	45.5 ± 3.9 (48)	46.9 ± 5.3 (54)
Gender, M : F	11 : 4	9 : 6	5 : 10
Diagnosis, number (%)			
Trauma	7 (47)	3 (20)	3 (20)
Burn	2 (13)	6 (40)	4 (27)
Ulcer	2 (13)	1 (7)	3 (20)
Cutaneous tumors	0 (0)	4 (27)	0 (0)
Others	4 (27)	1 (7)	5 (33)

### Platelet isolation

At the initiation of surgery, an 8.5 cc blood sample was collected from a vein in the upper arm without IV perfusion by means of a RegenKit® (RegenLab, En Budron B2, 1052 Le Mont-sur-Lausanne, Switzerland). The blood sample tube was immediately centrifuged for eight minutes at 2,800 rpm leading to a separation of the red cells from the plasma. Under these conditions, the platelets formed a narrow white band at the interface between the red cells and the plasma. After resuspending the platelets in the plasma by gentle agitation, the resulting platelet concentrate was transferred to another sterile tube and placed at 37°C. The final volume of platelet concentrate ranged between 3.5 and 4.5 ml. PC were controlled for cell type and concentration: some isolated mononuclear cells were observed

### Autologous keratinocyte isolation

A sample of 0.2 mm thick skin collected by means of a dermatome was placed in a transport tube containing 20 ml of DMEM, 10^2^ U/ml of penicillin and 0.1 mg/ml of streptomycin sulfate. The skin sample was sent along with the platelet concentrate to our cell culture laboratory. After being rinsed three times with an isotonic PBS solution, the skin sample was cut into small fragments (approximately 1 mm^2^) with a scalpel and digested in a 0.05% trypsin - 0.01% EDTA mixture for 45 minutes at 37°C. The supernatant containing the keratinocytes was centrifuged for three min at 200 g and the resulting cell pellet was washed twice in DMEM solution. The cells were then counted under the microscope. Finally, the cell pellet was suspended in 3.5 to 4.5 ml of autologous platelet concentrate. All procedures were conducted under a laminar flow, following strict rules of asepsis and sterility in a cell processing center, according to the GMP procedures.

### Surgical treatment

After collecting skin grafts, all donor sites were temporarily covered with gauze impregnated with an adrenaline solution (2 mg/L in saline). At the end of the surgical intervention, the PC group received a platelet concentrate obtained using the procedure described above. A spray applicator consisting of two syringes maintained at 37°C, one containing PC (10 volume) and the other a 10% calcium chloride solution (1 volume), was used to dispense the mixture on to the wound bed. The mixtures were left to coagulate *in situ* before applying any dressings. Mean cell counts in suspension for 4 ml were 2,300 × 10^6^ platelets and 22.6 × 10^6^ keratinocytes. Average counts on wounds were 13.5 × 10^6^/cm^2^ platelets and 80,000 keratinocytes/cm^2^ respectively. The PC + K group received a suspension of keratinocytes in PC following a similar procedure. Wounds from all groups were then covered with three layers of paraffin gauze (Jelonet®, Smith & Nephew, 15 Adam Street, London WC2N 6LA, UK), wrapped with standard bandages (Kerlix®, Covidien, 15 Hampshire Street, Mansfield, Ma) and contained in an elastic bandage. Control wounds were covered by three layers of paraffin gauze and bandages (the standard treatment for skin graft take in our department). All patients and observers were blinded to the treatment.

### Wound healing

Treatment efficiency was evaluated with respect to the duration (in days) until complete healing occurred along with assessments of the degree of epithelialization. The first evaluation was done on post-operative day five by an observer blinded to the type of treatment. The dressing and bandages were replaced with sheets of paraffin gauze and covered with standard dry dressings. Subsequent evaluations were done at two-day intervals until complete healing. Time until complete wound healing was recorded. Local pain, in general and specifically while replacing dressings, was evaluated by the patients on post-operative day five using a visual analogical scale with 0 representing no pain and 10 representing extreme pain. Both pre-operative and post-operative pictures documenting wound healing progression were also taken. Eventual adverse effects were to be recorded throughout the follow-up.

### Statistical analysis

Results are expressed as mean ± SEM. One-way ANOVA and *post hoc* Tukey’s tests were used to confirm statistically detectable differences between groups with *P*-values below 0.05. The statistical package was JMP® (Version 5.5, SAS Institute Inc., Cary, NC, USA).

## Results

Altogether, 45 patients were included (Table 1). There were no statistically significant differences between the groups with respect to gender and age despite the PC + K group having a ratio of female to male patients which was almost the inverse of that in other groups. The four most frequent pathologies in our groups were traumatic defects, burns, ulcer of venous etiology and cutaneous tumors. In the control group, the other categories consisted of one lumbar hemolymphangioma, one equinous deformity with neurinoma, one excision of a thigh sarcoma and one defect after total knee arthroplasty. In the PC group, there was only one patient outside the aforementioned categories. This case was a carcinoma of the rectum needing skin graft after perineal reconstruction. In the PC + K group, there were five patients not belonging to the main categories. These consisted of one hidradenitis suppurativa, one thigh sarcoma, one necrotizing pancreatitis, one ankle osteitis with secondary flap reconstruction and one keloid excision.

**Figure 1:**
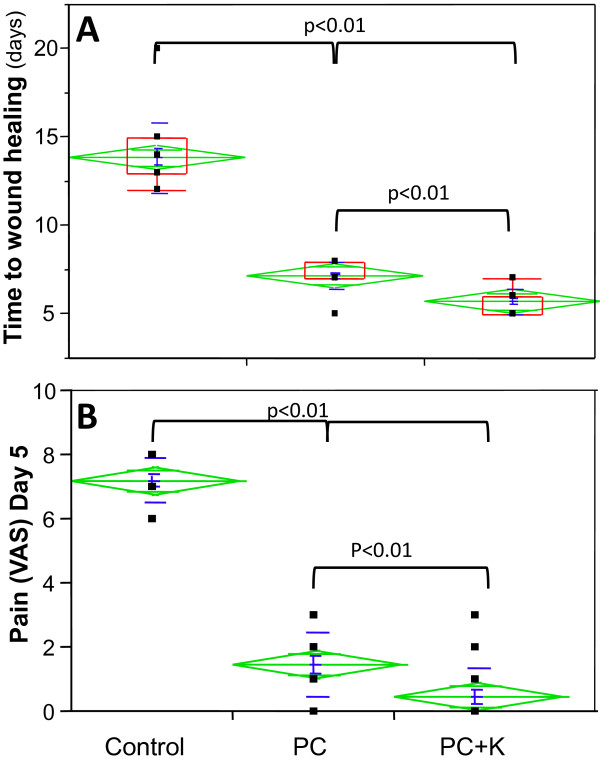
**Results show a significant reduction in time to wound healing associated with less pain.** (**A**) Duration in days until complete wound healing. (**B**) Pain intensity reported on post-operative day five using a visual analogical scale. Data as box plots (sample minimum, lower quartile, median and upper quartile as well as sample maximum).

**Figure 2:**
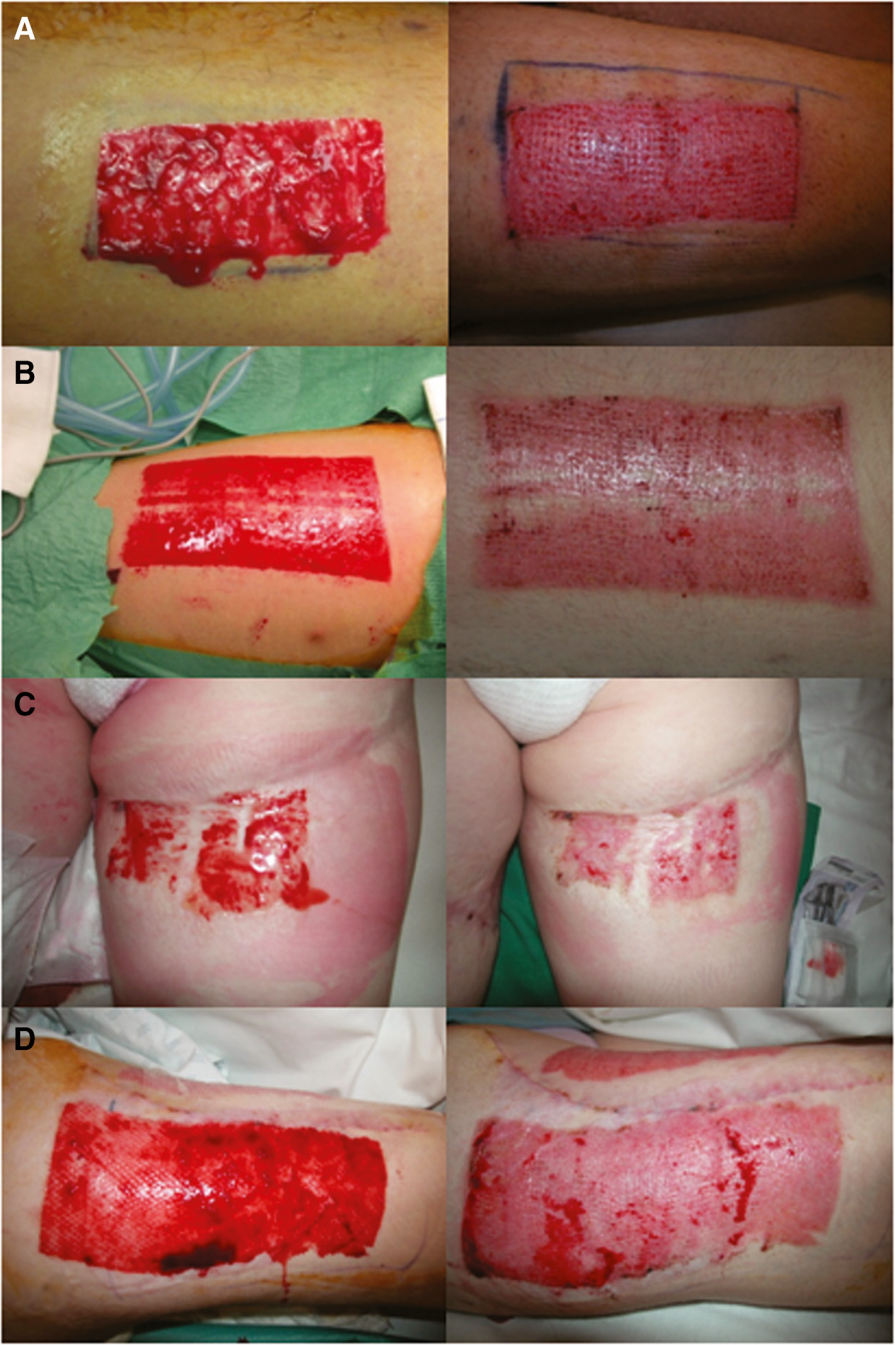
**Pictures demonstrate greater amount of epithelialization when treated with platelet concentrate or keratinocytes suspended in a platelet concentrate.** (**A**) and (**B**) show treatment with PC at operation time (left panel) and seven days later (right panel). (C) and (D) show treatment with PC + K at operation time (left panel) and five days later (right panel).

Wound healing was significantly shortened in PC and PC + K groups compared to control (*P* < 0.01), reflecting the stimulation of wound healing by the platelet concentrate (Figure 1A). In the PC group, wound healing time was reduced to 7.2 ± 0.2 days compared with 13.9 ± 0.5 days in the control. A further significant reduction of the healing time was observed in the PC + K group compared to the PC group (*P* < 0.01). Indeed, the PC + K group showed the fastest wound healing, averaging 5.7 ± 0.2 days (Figure 2).

Patients from the PC group also experienced a significant reduction of the pain resulting from the skin graft donor sites compared to the control group, an effect further enhanced in the PC + K group (Figure 1B).

No infection was noted. The tolerance and acceptance of the treatments were excellent and no undesirable effects were noted.

## Discussion

Studying clinical wound healing is challenging because of an inherently high variability, which may alter the evaluation of results. The principal differences lie within the wound itself, specifically its depth, localization, etiology and other associated pathologies [24]. This motivated our aim to evaluate skin graft donor sites rather than the grafted areas in an attempt to minimize inherent differences between wounds. Indeed, a skin graft remains the most used technique for wound closure [25,26] and the surgical technique has been refined over the years due to improvement of the apparatus used to take skin grafts. This now allows for an increased precision and reproducibility. For this reason and due to the relative ease of monitoring donor sites, this type of wound has become the ideal model to study wound healing *in vivo*[26]. Healing of the donor site, however, may vary and depends on both the depth of the wound and its surface area which may range from a few square centimeters to several hundred. The energy cost of healing is elevated and aggravated by losses of minerals, trace elements, proteins and fluids [25,26]. This becomes particularly important when extensive surfaces are involved, as in the case of major burns, major trauma, and in children and elderly patients. In those cases, new large wounds appear at skin graft donor sites, with an elevated risk of complication such as infections and delayed healing which further increases the toll on the body. It is therefore, extremely important to attempt to stimulate and accelerate donor site wound healing [27].

Under normal circumstances, and according to the inclusion criteria we defined, the graft donor sites investigated in this study should heal spontaneously within 12 to 14 days. Encouraging results were obtained while assessing the healing effect of the application of an autologous platelet concentrate alone and the application of a keratinocyte suspension combined with an autologous platelet concentrate to the donor site compared to a standard treatment. Indeed, clinical efficacy was notably enhanced as experimental groups showed an important reduction of healing time, particularly in the PC + K group which only required an average of five days, less than half the time required by conventional methods (Figure 1A). These results show the ability of concentrated platelets and keratinocytes to amplify and enhance the first phases of the healing cascade. In our trial, we didn’t include a group of patients receiving keratinocytes only, because previous work has already demonstrated its benefit on wound healing [28-31] and because our purpose was to verify the use of PC as a transport medium for keratinocytes.

Our study also confirms that platelet concentrates may be successfully obtained through a one-step isolation procedure. The protocols used in this study however, may of course be further optimized. For instance, although the platelet concentrates were prepared during the surgical intervention, it may be possible to do so on the eve of the operation, thereby effectively reducing the workload in the operating room. Similarly, we are currently investigating ways to further simplify methods of obtaining cutaneous cell suspensions and to better understand their contribution to enhancing the healing cascade and their interaction with the delivered platelets.

Different types of dressings such as hydrocellular, hydrocolloid, alginates and paraffin gauze have been tested in our department during the past few years in an attempt to reduce pain. Although subsiding with the use of some hydrocellular dressings, pain and discomfort almost always remained a problem during dressing replacement which is done imperatively in the first 48 hours and a week following the procedure [3,9,32]. The elevated cost per unit of the above mentioned dressing however becomes consequential relative to the wound surface to be covered [9,33,34].

The study shows significant pain reduction when using PC alone, and an even more pronounced reduction using the combination of PC with keratinocytes (Figure 1B). The effect was virtually immediate and long lasting and without the need for repeated application. Some patients who had previously healed skin graft donor site wounds noted the difference in treatment and found the recovery relatively pain free. This admittedly unforeseen observation could be partially explained by the ability of the platelet gel to maintain a humid environment around the wound. Different kinds of new dressings (for example, hydrofibers and hydrocolloids), however, attempt to generate that same effect although the pain does not disappear completely and is usually only attenuated, hinting that there may be more to the story. Indeed, platelets may be releasing or stimulating the release of substances with an analgesic effect. As shown previously [35], platelets are known to release endorphins, which may explain why the analgesic effect observed was rapid and long lasting. We have noted the same observation in patients presenting chronic wounds and treated with platelet gels (data not shown). Specific studies must be conducted to explain precisely how the platelet concentrates act directly or indirectly on pain in acute wounds and how keratinocytes may further enhance that effect, as we have seen in this study.

The risk of wound infection is directly related to the healing time, while delayed wound healing may in certain cases be the only sign of infection [9,10,36-38]. This can be explained by the bacterial release of endotoxins that increase pro-inflammatory cytokines such as Il-1 and TNF-α, thereby causing increased levels of metalloproteinases (MMPs) and a decrease in the production of growth factors [36]. Additionally, wound infections increase exudates and pain and are associated with increased patient discomfort, an unesthetic scar and an important increase in the cost of care. As such, infection of the donor site may sometimes expand, especially in debilitated, malnourished, polytraumatized patients, major burn victims or patients treated with drugs that render the skin fragile, such as corticoids. Ranging from cellulitis to septicemia, complications may very well threaten the patient’s survival.

No infectious complication was observed in this study. This can be explained by the acceleration of healing and the potential antibacterial role of platelets and white blood cells trapped in the platelet concentrate [39,40]. Additional studies may shed some light onto the mechanisms underlying this effect and whether platelets act directly to prevent infections or through the stimulation or other cells. Whether cutaneous cell suspensions help enhance this effect would also be of interest, especially in the case of chronic wounds.

The methods presented here benefit from an important advantage in that no heterologous product was used. PC can be obtained by simple centrifugation with a Conformité Européenne (CE) approved device. This study also demonstrates that platelet concentrate may be an excellent vehicle for transporting a suspension of autologous keratinocytes onto a wound. Keratinocytes, which play an essential role in wound healing thanks to their multiplicative capacity and their metabolic role [11-17], effectively enhance the effect of PC, an observation which opens the door to a much larger range of applications involving the addition of other cell types such as fibroblasts, which also play an important role in wound healing [7]. In the case of major burns, every day gained in terms of healing translates into increased chances of survival, reduced suffering, reduced scar ransom and an important decrease in what is usually a major cost to health institutions. The preparation of a PC requires a cell processing unit and qualified personnel for cell isolation. The laboratory procedure takes approximately 90 minutes. Taking into account the personnel salary and the kit for platelet isolation, the cost of a PC + keratinocyte preparation may be estimated between 300 to 500 Swiss Francs. There is no major manipulation of the tissue, including cell culture and expansion. As only enzymatic treatment and centrifugation steps are needed, tissue can be rapidly processed in a Class A laminar flow system dedicated for this activity.

## Conclusion

The data show that platelet concentrates have a beneficial effect on wound healing and can be used to deliver keratinocytes to the wounds. The association of platelet concentrates and keratinocytes appears to be the most efficient way to enhance wound healing. We believe that this method could be the first line treatment for patients with impaired healing. However, the complexity and cost of the method have to be reduced in order for this technique to be used as a standard protocol.

This study showed beneficial effects regarding pain and time to complete wound healing. This procedure opens the path towards a more efficient treatment of chronic wounds such as ulcers, including decubitus ulcers and those related to diabetic neuropathies or necrotic angiodermatitis. It also provides undeniable advantages in treating major burn victims. Future trials are required to address the question of the specific impact of keratinocytes on wound healing.

## Consent

Written informed consent was obtained from the patient for publication of this report and any accompanying images.

## Abbreviations

ANOVA: Analysis of variance; CE: Conformité Européenne; DMEM: Dulbecco’s modified Eagle’s medium; EDTA: Ethylenediaminetetraacetic acid; EGF: Epidermal growth factor; FGF: Fibroblast growth factor; GMP: Good manufacturing practice; IL-1: Interleukin-one; MMPs: Matrix metalloproteinases; PBS: Phosphate buffered-saline; PC: Platelet concentrate; PC + K: Platelet concentrate + Keratinocytes; PDGF: Platelet derived growth factor; SD: Standard deviation; SEM: Standard error of the mean; TNF-α: Tumor necrosis factor- alpha.

## Competing interests

The authors have nothing to disclose regarding sources of financial support or commercial sponsorship of the work described in this manuscript.

## Authors’ contributions

SG participated in inclusion of patients, data collection, and statistical analysis and drafted the manuscript. SD participated in the process of writing the article and analyzing the data. MMB made the statistical analysis and reviewed the whole manuscript. MB made the laboratory work and helped write the text. WR designed the study, included and followed-up the patients and reviewed the manuscript. All authors read and approved the final manuscript.

## Meetings at which this work’s data has been previously presented

1 – Oral Presentation, Chirurgie Plastique et Reconstructive Research Day, Centre Hospitalier Universitaire Vaudois, November 2008.

2 – Oral Presentation, 35th Annual meeting of European Society for Artificial Organs. Geneva, September, 2008.

3 – Oral Presentation, 19th EURAPS Meeting (European Association of Plastic Surgeons), Madeira, May 2008.

4 – Oral Presentation, 43rd Congress of SSCPRE (Société Suisse de Chirurgie Plastique, Reconstructive et Esthétique), Montreux, October 2007.
